# Surviving a flood: effects of inundation period, temperature and embryonic development stage in locust eggs

**DOI:** 10.1017/S0007485315000243

**Published:** 2015-04-01

**Authors:** J.D. Woodman

**Affiliations:** Australian Plague Locust Commission, Australian Government Department of Agriculture, GPO Box 858, Canberra 2601, Australia

**Keywords:** insect, survival, *Chortoicetes terminifera*, rainfall, population dynamics

## Abstract

The Australian plague locust, *Chortoicetes terminifera* (Walker), is an important agricultural pest and oviposits into compacted soil across vast semi-arid and arid regions prone to irregular heavy summer rainfall. This study aimed to quantify the effects of flooding (control, 7, 14, 21, 28 and 35 days) at different temperatures (15, 20 and 25°C) and embryonic development stages (25 and 75%) on egg viability, hatchling nymph body mass and survival to second-instar. Egg viability after flooding was dependent on temperature and flood duration. Eggs inundated at 15°C showed ≥53.5% survival regardless of flood duration and development stage compared with ≤29.6% for eggs at 25°C for ≥21 days early in development and ≥14 days late in development. Hatchling nymphs did not differ in body mass relative to temperature or flood duration, but weighed more from eggs inundated early in development rather than late. Survival to second-instar was ≤55.1% at 15 and 20°C when eggs were flooded for ≥28 days late in development, ≤35.6% at 25°C when flooded for ≥28 days early in development, and zero when flooded for ≥21 days late in development. These results suggest that prolonged flooding in summer and early autumn may cause very high egg mortality and first-instar nymph mortality of any survivors, but is likely to only ever affect a small proportion of the metapopulation. More common flash flooding for ≤14 days is unlikely to cause high mortality and have any direct effect on distribution and abundance.

## Introduction

For terrestrial animals that inhabit environments prone to irregular heavy rainfall and the potential for flooding, the capacity to endure variable periods of inundation at different times of year is critical to survival. Many invertebrate species that exploit relatively frequently inundated shoreline or floodplain habitats show specific behavioural, reproductive, phenological and physiological adaptations. For example, to avoid drowning mobile life stages may horizontally or vertically migrate to non-flooded sites (e.g., earthworms, Plum, 2005; Zorn, *et al.*, 2008), and sedentary life stages may enter a quiescent state (e.g., eggs of the collembolan, *Isotoma viridus*, Tamm, 1986; larval tiger beetles, *Cicindela togata*, Hoback *et al.*, 1998). Conversely, species that inhabit less frequently inundated environments with high climatic variability and rainfall irregularity may be more susceptible to mortality from flooding (Adis & Junk, 2002; Plum, 2005). Indeed, survival in the latter scenario may be the most marginal for sessile life stages and species that inhabit holes or cracks in soil with limited capacity for behavioural response. Extreme rainfall events leading to flooding can therefore have major impacts on patterns of distribution and abundance (Morton *et al.*, 2011).

**Fig. 1.**
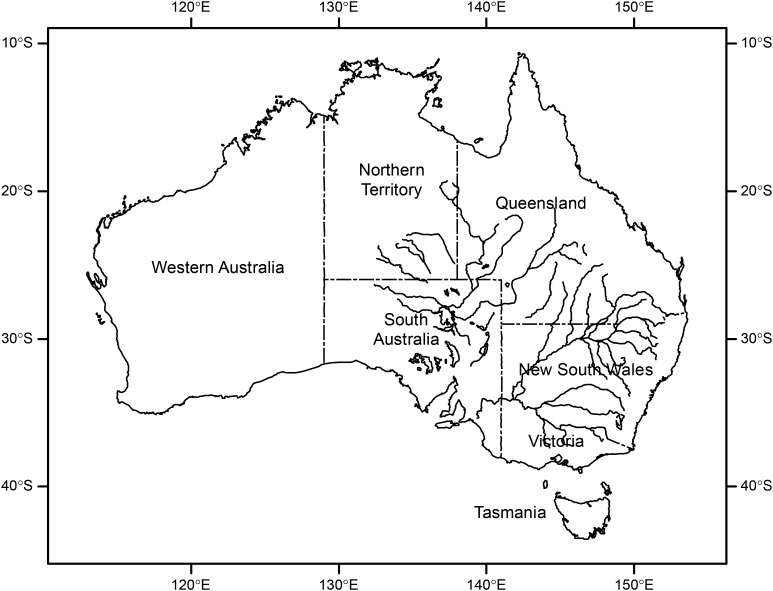
Map of inland river and lake systems of the Lake Eyre and Murray–Darling River catchments in mostly arid and semi-arid regions of Eastern Australia. Intense rainfall events typically associated with summer storms can rapidly lead to flooding. The entire area encompassing both systems provides favourable habitat for the Australian plague locust, *Chortoicetes terminifera*.

In Australia, vast semi-arid and arid inland areas are subject to high rainfall variability and sudden transitions between short but intense wet periods and long dry periods (Hughes, 2003; Pittock *et al.*, 2006; Morton *et al.*, 2011). This rainfall variability and the likelihood of flooding events are influenced by phases of the El Nino-Southern Oscillation in Eastern Australia as well as the Indian Ocean Dipole and Southern Annular Mode (King *et al.*, 2013). Flood frequency, duration and magnitude are also dependent on other factors including run-off dynamics and localized regulation of downstream water courses (e.g., Boulton & Lloyd, 1992). Flooding in Australia is generally either flash flooding of relatively short duration and limited spatial spread from localized heavy rainfall, or more widespread basin flooding along expansive drainage systems (fig. 1). Basin flooding events also often move hundreds of kilometres downstream before abating (Pittock *et al.*, 2006). While localized inland flash flooding occurs relatively frequently, dependent on the distribution and intensity of heavy rainfall, basin flooding is less common, but increased in frequency in the eastern half of the continent over the second half of the last century (Hughes, 2003). Further increases in summer storm activity and frequency of flooding events are possible with anthropogenic climate change (Whetton *et al.*, 1993; Hughes, 2003; Hirabayashi *et al.*, 2013).

The Australian plague locust, *Chortoicetes terminifera* (Walker), regularly inflicts agricultural damage owing to it being well adapted to climatic and habitat variability and consequently occupying a broad distribution that spans the entirety of the inland of the continent. There are typically two to four generations per year dependent on adequate rainfall during Spring, Summer and Autumn providing sufficient soil moisture for vegetation growth and embryonic development (Hunter *et al.*, 2001). Eggs of *C. terminifera* are deposited into soil at a depth of 2–10 cm in froth-lined pods typically containing 30–60 eggs that may exceed 1000 m^−2^ during swarm oviposition events (Australian Plague Locust Commission (APLC), unpublished data). Individual egg pods typically show high hatching synchrony whereby an initial flush of most of the eggs within 24 h is followed by several days of occasional nymph emergence. The eggs exhibit a facultative diapause mechanism for overwintering as well as two identified quiescence points during embryonic development in response to low soil moisture (water is not available to the eggs if held by the soil with a suction force > 9–10 atm) (Wardhaugh, 1973, 1980*a*; Deveson & Woodman, 2014*a*). Although the effects of insufficient rainfall and environmental moisture in decreasing population size are relatively well understood (Clark, 1974; Hunter *et al.*, 2001), the potential for excessively wet conditions to have similar effects are not. In particular, the potential for soil flooding after intense rainfall events associated with the passage of tropical low-pressure systems in summer to cause egg mortality remains unknown (Pickford, 1966; Brust *et al.*, 2007; Woodman, 2013). Given that relatively poor draining clay pan soils are favoured *C. terminifera* oviposition sites and can be particularly prone to flooding (Clark, 1974), this study was designed to quantify the effects of different inundation periods on locust egg viability, hatchling nymph mass and subsequent survival to second-instar.

## Materials and methods

### Insects

Gravid adult female *C. terminifera* were obtained from a gregarious captive colony maintained at the University of Sydney, Australia. All individuals were kept at a L14:D10 photoperiod at 38 ± 0.5 and 28 ± 0.5°C, respectively, in the laboratory, which results in negligible diapause egg production (Wardhaugh, 1980*b*; Deveson & Woodman, 2014*a*, *b*). The locusts were housed in crowded conditions in square enclosures of aluminium frame and fibreglass mesh construction (dimensions: 30 × 30 × 30 cm) and provided with freshly cut wheat seedling leaves as food *ad libitum* to permit fat accumulation necessary for oocyte development.

### Soil preparation and locust oviposition

To provide a suitable natural substrate for *C. terminifera* oviposition, red-brown earth topsoil (A horizon) was collected to a depth of approximately 15 cm from an egg bed site near Griffith, in the Riverina region of New South Wales (34°07.913S, 147°23.721E). Soil particle fractions were 35.2% coarse sand, 43.6% fine sand, 5.1% silt and 16.1% clay, with an electrical conductivity (EC) of 39.2 uS cm^−1^ and a pH of 5.4 (HI98312, Hanna instruments, USA). This soil type is typical of *C. terminifera* oviposition sites in Southeastern Australia and egg pods had been previously collected at the site. The soil was returned to the laboratory and manually crushed to break up any large clods before being dried at 70°C for 24 h. The dry soil was then moistened with filtered tap water (QL1-Zip 5 μ water filter, Zip industries, Sydney, Australia) to 8% water by weight (sensitivity 0.01 g, PB3002-L, Mettler-Toledo, USA) and firmly packed to a depth of approximately 8 cm into small cardboard pots with a waxed internal surface to ensure water-holding integrity (internal dimensions: diameter = 74 mm (top) and 54 mm (bottom), height = 85 mm, volume = 237 ml). To allow for oviposition, individual gravid female locusts were placed on the soil surface inside each container covered with fibreglass mesh before being returned to the incubator set as above. After each observed oviposition (i.e., one egg pod per female per container) the container was removed from the incubator and the locust was removed. Containers with confirmed oviposition were then grouped to give *n* = 7–9 egg pods per treatment.

### Flooding experiments

Treatment groups consisted of 0 (control), 7, 14, 21, 28 and 35-day flooding periods at 15, 20 and 25°C for direct developing (i.e., non-diapause) egg cohorts at 25 and 75% development time (embryonic development rates with temperature are given in Wardhaugh, 1973). For each treatment, incubation temperature before and after flooding was maintained at 23, 28 and 33°C (i.e., 8°C higher than the respective flooding temperature) to approximate changes in soil temperature associated with heavy rainfall events recorded at a depth of 10 cm in the field (APLC, unpublished data). The 25 and 75% embryonic development stages were selected to represent different phases of development progression and correspond with early (pre-anatrepsis) embryonic development, and late embryonic development (where the embryo has filled the entire egg space) (Wardhaugh, 1973). Inundation was achieved by saturating the soil with filtered tap water to a point where the water level exceeded the soil surface by 10–15 mm. Soil in each of the control groups was maintained at 8% water by weight content.

At the end of each flooding period, the excess surface water was drained off each cup and ten small holes of 1 mm diameter were pierced in the base to allow drainage. Each cup was then placed on an aluminium tray of soil to collect the draining water and returned to the respective incubator. A second group of control cups were flooded without any egg pods (*n* = 6 per temperature treatment) and weighed daily from the time of draining for up to 6 days to determine comparative soil-drying rates, which were 9.23 ± 0.57 g day^−1^ at 23°C, 10.72 ± 1.37 g day^−1^ at 28°C and 14.16 ± 1.22 g day^−1^ at 33°C. All the cups containing egg pods were checked for hatching and weighed to monitor soil moisture content daily. Soil moisture was kept at 8% by adding additional water to compensate for evaporation and drainage as necessary.

Within 24 h of hatching, nymphs from each egg pod were removed, counted and weighed (sensitivity 0.1 mg, AB104-S, Mettler Toledo, USA) before being transferred into separate 800 ml plastic containers with fresh wheat seedling leaves provided *ad libitum*. Nymphs were incubated using a L14:D10 photoperiod at 36 ± 0.5 and 28 ± 0.5°C, respectively, to quantify survival to second-instar. Nymphs hatching from control egg pods were treated the same way as the flooded eggs to quantify hatching success, hatchling nymph body mass and survival to second-instar without inundation.

Egg pod excavation and dissection was performed 7 days after the last hatching within an egg pod per treatment set. The numbers of dead and unhatched eggs as well as dead and live trapped nymphs were recorded. Nymphs that had hatched but not emerged (either still alive or recently dead) from the soil were weighed and treated as hatched nymphs.

### Data analysis

Data were analysed using generalized linear models. Percentage survival data were arcsin transformed prior to analysis. All statistical procedures were performed using Minitab (v. 14, Minitab Inc., USA).

## Results

Egg survival was significantly decreased by increased flood temperature (d.f. = 2, *F* = 16.86, *P* < 0.001) and by increased flood duration (d.f. = 5, *F* = 6.08, *P* < 0.001) (table 1). A significant interaction whereby increased temperature and increased flood duration resulted in greater mortality was apparent (d.f. = 10, *F* = 2.20, *P* = 0.019), but interaction for increased temperature and development stage at inundation was marginally non-significant (d.f. = 2, *F* = 2.75, *P* = 0.066). Interaction for flood duration and development was also non-significant (d.f. = 5, *F* = 1.22, *P* = 0.300). Eggs inundated at 15°C had ≥53.5% survival regardless of flood duration and development stage compared with eggs inundated at 25°C, which had ≤29.6% survival when flooded ≥21 day early (25% of total embryonic development) in development and ≥14 day late (75% of total embryonic development) in development (table 1). A small number of unexpected whole egg pod failures were observed across the treatment groups where a mixture of dead nymphs and eggs were found trapped under the soil surface. These were retained for analysis and corresponded to 8, 5 and 3 egg pods at the 15, 20 and 25°C flooding temperatures, respectively. These egg pods were distributed across different flooding durations (i.e., not clustered) from treatments that otherwise showed > 50% total egg survival.

**Table 1.** Survival data (%) for *Chortoicetes terminifera* eggs to the point of hatching and nymph emergence following flooding (except controls (C)) of whole egg pods (*n* = 7–9 with the total number of eggs per treatment given in parentheses) at early (E) or late (L) in development (see Materials and methods section) *in situ* in soil at 15, 20 and 25°C.

Flood duration (day)	C 15°C	E 15°C	L 15°C	C 20°C	E 20°C	L 20°C	C 25°C	E 25°C	L 25°C
0	75.2 (298)			87.9 (355)			98.6 (345)		
7		80.4 (314)	75.4 (317)		86.9 (336)	79.5 (269)		83.9 (323)	62.9 (321)
14		67.4 (331)	78.4 (329)		75.9 (278)	77.5 (276)		70.5 (352)	18 (327)
21		69.2 (347)	66.2 (340)		76 (312)	68.7 (358)		28.9 (384)	29.6 (324)
28		61.1 (355)	66.6 (323)		79.7 (309)	51.8 (329)		26.5 (339)	0 (346)
35		73.5 (321)	53.5 (316)		88.7 (302)	33 (316)		24.3 (313)	0 (349)

Hatchling nymph survival was significantly decreased by increased flood temperature (d.f. = 2, *F* = 15.95, *P* < 0.001) and by increased flood duration (d.f. = 5, *F* = 23.84, *P* < 0.001) (table 2). Significant interactions whereby increased temperature and increased flood duration (d.f. = 10, *F* = 3.59, *P* < 0.001), and increased flood duration and later development stage at inundation (d.f. = 5, *F* = 9.03, *P* < 0.001) resulted in greater mortality were apparent. Interaction for temperature and development stage was non-significant (d.f. = 2, *F* = 1.80, *P* < 0.168).

**Table 2.** Survival data (%) for *Chortoicetes terminifera* nymphs to the point of successful moult to second-instar that hatched following flooding (except controls (C)) (total number of nymphs per treatment given in parentheses) during embryonic development at early (E) or late (L) development stage *in situ* in soil at 15, 20 and 25°C.

Flood duration (day)	C 15°C	E 15°C	L 15°C	C 20°C	E 20°C	L 20°C	C 25°C	E 25°C	L 25°C
0	85.7 (224)			89.7 (312)			85.6 (340)		
7		91.6 (238)	90.8 (239)		90.1 (284)	88 (215)		93 (271)	92.1 (202)
14		95.1 (223)	91.1 (258)		91.1 (201)	84.8 (237)		92.7 (248)	83.1 (59)
21		82.5 (240)	67.6 (225)		84 (231)	73.5 (236)		90.1 (100)	52.1 (90)
28		85.7 (217)	27 (215)		88.3 (237)	55.1 (190)		35.6 (90)	0 (0)
35		86.9 (236)	26.6 (169)		86.3 (275)	45.8 (73)		69.7 (76)	0 (0)

Hatchling nymph body mass was 4.8 ± 0.4–5.6 ± 0.4 mg among all treatment groups. It did not differ relative to flood temperature (d.f. = 2, *F* = 2.39, *P* = 0.095) or duration (d.f. = 5, *F* = 0.77, *P* = 0.575), but was higher for eggs inundated early in development (d.f. = 1, *F* = 6.03, *P* = 0.015). Nymph survival was ≤55.1% at 15 and 20°C if eggs were inundated for ≥28 days late in development, ≤35.6% at 25°C if inundated for ≥28 days early in development, and zero survival if inundated for ≥21 days late in development (table 2).

## Discussion

Flooding can exert strong influence on the distribution and abundance of soil invertebrates, particularly in arid regions where flooding events are sporadic and water accumulation and drainage variable (Hunter *et al.*, 2001; Adis & Junk, 2002; Plum, 2005). The laboratory experiments reported here show that direct developing eggs of *C. terminifera* are resilient to short-term flooding events (≤14 days) irrespective of soil temperature and embryonic development stage at inundation. However, when flooded for longer periods, egg survival depends on temperature. For example, survival from ≥21 days flooding was strongly temperature dependent, whereby at 15°C there was relatively little change in egg mortality at longer durations, but at 25°C mortality dramatically increased, especially for eggs inundated late in development (table 1). Thus, non-diapause *C. terminifera* eggs appear to have some physiological capacity to withstand flooding (at least at cooler temperatures), which is not surprising given that at least some locust egg beds are likely to be flooded most years in Southeastern Australia. Additionally, high reproductive rates, a widespread distribution and the potential for re-immigration into areas following catastrophic events by long-distance migration may provide further population resilience in response to transient, regional population bottlenecks (Clark, 1974; Hunter *et al.*, 2001; Chapuis *et al.*, 2011).

The findings of this study are consistent with those previously reported for *C. terminifera* by Hogan (1965), where control survival of 87% decreased to 83, 58, 19 and 8% after 16, 30, 40 and 50 days for individual eggs submerged directly into water at 17°C and then transferred to moist sand at 26°C. However, these data are not directly comparable due to a different temperature regime, no record of whether the eggs used were direct developing or in diapause (or the embryonic development stage), and the direct submersion of individual eggs. Among other acridids, Ackonor (1989) reported that eggs of *Locusta migratoria migratorioides* flooded just after oviposition or just before hatching were most susceptible to mortality and that embryonic development times were retarded by flooding. However, a single constant experimental temperature of 34 ± 1°C was used based on optimum non-flooded embryonic development temperatures that would represent an unlikely inundation temperature in the field, and resulted in complete mortality for eggs flooded for >3 days (Ackonor, 1989). Ting *et al.* (2009) quantified the effects of flooding on egg survival in the tropical grasshopper, *Fruhstorferiola tonkinensis*, at low temperatures (15–21°C) and reported a marked decrease in survival after flooding for ≥15 days and no survival at 30 days. This species therefore appears to be less tolerant of inundation than *C. terminifera*, despite it being a known pest of rice and therefore potentially experiencing an increased likelihood of encountering flooded soil. However, a two-stage temperature regime was not used and the development stage of the eggs at inundation was not reported. Most strikingly, Stauffer *et al.* (2011) reported that 64% of eggs of the grasshopper, *Romalea microptera*, survived 90 days of submersion in sand at a relatively high 24–28°C. However, this species has a very different life history compared with *C. terminifera* (e.g., longer development times, larger size) and inhabits salt marsh that is subject to seasonal flooding for extended periods that would be exceedingly rare in *C. terminifera* oviposition habitat.

This study did not investigate possible differences in the effects of flooding on eggs in diapause compared with direct development, but previous studies on other acridids have suggested greater tolerance to flooding in diapause eggs, and have reported no increase in egg mortality from winter flooding events (Richards & Waloff, 1954; Ting *et al.*, 2009; review: Adis & Junk, 2002). The most extreme known survival example reported is for *Locusta migratoria* in China where water submerged diapause egg pods in the field remained viable for up to 8–9 months (compared with ≤30 days for direct developing eggs at 30°C in the laboratory) if oviposited in early autumn (Chin *et al.*, 1959). In floodplain grasslands of the Lower Oder River in Europe, the long-horned grasshopper, *Metrioptera roeseli*, and short-horned grasshoppers, *Chorthippus brunneus* and *C. parallelus* are also apparently able to withstand flooding while in diapause (Adis & Junk, 2002). However, supporting empirical data is not shown and given the strong interactive effect of temperature and flood duration, it may be possible that metabolic rate is slowed sufficiently in direct developing overwintering eggs at low temperatures to confer similar tolerance to flooding. This is potentially relevant for *C. terminifera* egg beds which overwinter as a mixture of diapause and direct developing embryos, dependent on the cumulative effects of declining photophase (and to a lesser extent, temperature) defining a distinct temporal window of diapause oviposition (Wardhaugh, 1980*b*; Deveson & Woodman, 2014*a*). Given that *C. terminifera* embryonic development is known to reduce to negligible levels at soil temperatures of ≤13.5°C (Wardhaugh, 1973), it is plausible that flood tolerance may not be vastly different between diapause and direct developing eggs in a winter flood situation in more temperate southern habitat (e.g., >29°S latitude). However, in more northern habitat where soil temperatures remain higher during winter, diapause could confer some as yet unknown survival advantage. Importantly, differences between diapause and direct developing acridid eggs in the laboratory could arise as a result of exposing both to the same experimental temperature, which may be higher than that expected at appropriate soil depths under flood (particularly in winter), and artificially exacerbate the difference in metabolic rate between the groups (provided diapause is not broken) (see Ting *et al.*, 2009).

While the viability of the exposed life stage is likely to be the most important measure of survival relative to any environmental stress, survival of the subsequent life stage may reveal any on-going or delayed costs. For the resultant first-instar *C. terminifera* nymphs in the present experiments, hatchling mass and survival were quantified to determine any costs associated with embryonic development and emergence into a water saturated environment. While hatchling nymph body mass did not differ relative to temperature or flood duration, it was higher for eggs inundated early in development. The higher nymph mortality after late embryonic stage inundation lasting ≥28 days regardless of temperature could be due to lower body mass, but the causes require further research. It is possible that the hatchlings may have spent a longer period of time in a water saturated environment before emerging to the soil surface and could have suffered both directly from water contact and humidity as well as indirectly through increased exposure to dead conspecifics and the associated fungus/potential pathogens (see Hajek & St Leger, 1994).

In addition to the direct effects of water saturation on locust survival, flooding in arid and semi-arid environments can cause the erosion of topsoil and displacement of clay, silt, fine sand and organic matter to varying depths relative to the patchiness of vegetation and water flow patterns (Ludwig *et al.*, 2005; Bartley *et al.*, 2006). Indeed, at fine spatial and temporal scales, the displacement of topsoil fractions from heavy rainfall and flooding can be substantial and highly erratic (Ludwig *et al.*, 1999). Results from this study suggest that the viability of eggs and newly hatched nymphs of *C. terminifera* attempting to emerge to the soil surface is subject to relatively small changes to the soil surface, especially at lower temperatures when hatchling nymphs may be less physically able to excavate and push their way out onto the soil surface. Dissections of the failed egg pods indicated that the newly hatched nymphs lined up in single file beneath the surface may have especially limited capacity for pushing through the sediment layer if the soil has at least partially dried out and hardened. While the adverse effects on eggs of exposing egg pods through erosion or other physical means (e.g., tillage) have been previously observed (but not quantified), this study suggests that increased sediment over the top of the egg pod may cause whole egg pods to fail. This is despite newly hatched nymphs appearing to be able to remain in the crowded egg pod drill hole for at least 24 h prior to emerging to the soil surface without any apparent ill-effects (data not shown). While heavy summer storm rainfall has the potential to rapidly shift surface sediment, egg pods that overwinter (and therefore spend much longer time underground) are more likely to be subject to repeated displacements that could exert considerable influence on nymph emergence success, and this requires further research.

Whether considerable locust mortality can be caused by excessively wet conditions in the field has been a long-standing question for locust population monitoring and forecasting (Hogan, 1965; Ackonor & Vajime, 1995). In Australia, renewed interest has arisen owing to widespread wet inland conditions in 2010/2011 (King *et al.*, 2013), and more specifically, the sudden collapse of a major *C. terminifera* plague in late Spring/early Summer 2010 that coincided with the above average rainfall (Deveson & Woodman, 2014*b*). The results presented here report non-diapause egg survival after flooding using whole, intact froth lined egg pods oviposited into natural substrate collected from the field. This study highlights the temperature dependence of flood tolerance in addition to the potential importance of embryonic development stage at inundation for subsequent hatchling nymph survival. While prolonged flooding at high temperatures in summer and early autumn may cause very high egg mortality and subsequent first-instar nymph mortality of any survivors, it is likely to only ever affect a small proportion of the overall metapopulation. More common flash flooding for ≤14 days is unlikely to cause high mortality and have any direct effect on distribution and abundance in the field at any time of the year.
